# Impact of microRNA-34a and polymorphism of its target gene *CA9* on susceptibility to uterine cervical cancer

**DOI:** 10.18632/oncotarget.20842

**Published:** 2017-09-12

**Authors:** Shun-Fa Yang, Yu-Fan Liu, Chao-Wen Cheng, Wei-En Yang, Wea-Lung Lin, Jiunn-Liang Ko, Po-Hui Wang

**Affiliations:** ^1^ Institute of Medicine, Chung Shan Medical University, Taichung, Taiwan; ^2^ Department of Medical Research, Chung Shan Medical University Hospital, Taichung, Taiwan; ^3^ Department of Biomedical Sciences, Chung Shan Medical University, Taichung, Taiwan; ^4^ Graduate Institute of Clinical Medicine, College of Medicine, Taipei Medical University, Taipei, Taiwan; ^5^ School of Medicine, Chung Shan Medical University, Taichung, Taiwan; ^6^ Department of Pathology, Chung Shan Medical University and Chung Shan Medical University Hospital, Taichung, Taiwan; ^7^ Department of Obstetrics and Gynecology, Chung Shan Medical University Hospital, Taichung, Taiwan

**Keywords:** carbonic anhydrase, single nucleotide polymorphisms, miR-34a, migration, tissue microarray

## Abstract

The purposes of this study were to associate the genetic polymorphisms in carbonic anhydrase *(CA)* 9 with uterine cervical cancer and identify the clinical implications. Three single-nucleotide polymorphisms (SNPs), rs2071676 (+201, G/A), rs3829078 (+1081, A/G), and rs1048638 (+1584, C/A), and an 18-base-pair deletion/insertion (376del393) in *CA9* were examined. We used the Boyden chamber assay to evaluate the influence of *CA9* on the migration of cervical cancers. Tissue microarrays were used to evaluate CAIX immunoreactivity and determine its clinical significance. The results revealed that the *CA9* SNP rs1048638 is the only significant polymorphism that increases the risk of cervical cancer in Taiwanese women. We discovered that the *CA9* SNP rs1048638 influences the expression of *CA9* through the interaction between the 3′-untranslated region (UTR) of exon 11, where the SNP is located, and miR-34a, and influences the migration of cervical cancer cells. Moreover, we demonstrated that CAIX immunoreactivity is related to the occurrence of cervical cancer, and elevated CAIX immunoreactivity is associated with a more advanced stage. In conclusion, the finding that the *CA9* SNP rs1048638 exerts its action through duplexes of the miR-34a and *CA9* 3′-UTRs and plays a vital role in cervical cancer in Taiwanese women may be applicable to translational medicine.

## INTRODUCTION

Uterine cervical cancer is the fifth most common type of cancer in women in Taiwan. The Health Promotion Administration of the Ministry of Health and Welfare in Taiwan reported its age-standardized incidence rate in 2009 to be 11.86 per 100,000 women. Cervical cancer ranked seventh in cancer deaths in Taiwanese women in 2011. Preinvasive lesions associated with cervical cancer are defined as cervical intraepithelial neoplasia and are currently graded as low or high squamous intraepithelial lesions (LSIL or HSIL). Approximately 10% of LSIL and approximately 20%–30% of HSIL may progress to invasive cancer [1, 2].

The high metabolic rate of tumor cells often results in hypoxia and acidosis in poorly perfused regions; therefore, they develop the ability to function in a more acidic environment than normal cells [3–5]. To date, 13 active and 3 inactive isoforms of carbonic anhydrases (CAs) have been characterized in mammals, and most CAs can efficiently catalyze a reversible hydration reaction: CO_2_ + H_2_O D HCO_3_^−^ + H^+^ [6, 7]. In contrast to the other isoforms, the membrane-associated CAIX and CAXII isoforms are substantially increased in a hypoxic environment and are considered key pH regulators [6, 7]. CAIX is the most active isoform of CA in the hydration of carbon dioxide [8]. In response to hypoxia in human cancer cells, *carbonic anhudrase 9* (*CA9)* is the most strongly expressed gene [9, 10]. It is overexpressed in many tumors and is associated with cancer progression [11–16].

Single-nucleotide polymorphism is a difference in a single nucleotide in the shared sequence of a gene between the members of a species or paired chromosomes in an individual in at least 1% of a certain population [17]. SNP is probably associated with the development and occurrence of certain diseases such as cancers. When an SNP occurs in a coding sequence, it may change the encoded amino acids in the related protein and is known as nonsynonymous. If an SNP leads to the same amino acid, it is called a synonymous SNP. In addition, an SNP in the 3′-untranslated region (UTR) of a gene may influence biological processes [18]. *CA9* is located on chromosome 9p13–p12 and consists of 11 exons. It encodes for the 459-amino-acid protein CAIX [19]. Several studies have suggested that *CA9* variations influence the severity and prognosis of several types of cancer, including prostate cancer, urothelial cell carcinoma, and oral cancer [20–22].

According to our review of the relevant literature, few studies have correlated *CA9* SNPs with cervical cancer. We hypothesized that *CA9* SNPs have an impact on the expression of CAIX. Furthermore, *CA9* is overexpressed in many cancers [11–13]. Therefore, we investigated the distribution of the *CA9* SNPs rs2071676 (+201, G/A) in exon 1, rs3829078 (+1081, A/G) in exon 7, rs1048638 (+1584, C/A) in the 3′-UTR of exon 11, and an 18-base-pair deletion/insertion (376del393 in exon 1) among patients with cervical cancer and normal controls; moreover, we defined their clinical implications in Taiwanese women. We further delineated the mechanism by which *CA9* SNPs may influence the expression of CAIX in cervical cancer and associated the CAIX expression with the clinicopathological variables, cancer recurrence, and survival of patients with cervical cancer.

## RESULTS

The age distribution differed significantly between patients with cervical cancer and controls (53.9 ± 11.9 vs. 44.2 ± 10.2, *p* < 0.001). However, age difference was not correlated with the *CA9* SNP distribution (*p* = 0.181 for rs2071676, *p* = 0.758 for rs3829078, *p* = 0.191 for rs1048638, and *p* = 0.244 for 376del393). The genotype distributions of the SNPs rs2071676, rs3829078, and rs1048638 were in Hardy–Weinberg equilibrium (*p* = 0.406, χ^2^ value: 0.689; *p* = 0.745, χ^2^ value: 0.106; and *p* = 0.323, χ^2^ value: 0.976, respectively).

### Association between CA9 gene polymorphism and cervical cancer

A significant difference was observed in the distribution of the *CA9* SNP rs1048638 (*p* = 0.011) between women with cervical cancer and controls (Table 1). However, no such difference was observed in the distribution of rs2071676, rs3829078, and 376del393. The distribution of the CA/AA genotype of the *CA9* SNP rs1048638 differed between patients with cervical cancer and controls when the wild genotype CC was used as a reference (*p* = 0.007). After age was controlled for, women with the CA/AA genotype exhibited a higher risk (adjusted odds ratio [AOR]: 1.92, 95% CI: 1.01–3.65) of cervical cancer compared with those with the wild genotype CC. Although no significant difference was observed among the patients with *CA9* 376del393, notably, no patients with cervical cancer exhibited 376del393 in both chromosomes (deletion/deletion).

**Table 1:** Genotype distribution of single nucleotide polymorphisms of carbonic anhydrase 9 gene in patients with uterine cervical cancer or normal women

Variables	Control (n=327)	Cervical cancer(n=123)	*p* value	OR (95% CI)^b^	Adjusted OR(95% CI)^c^
**rs2071676**					
GG^d^	79 (24.2%)	26 (22.8%)	0.109	1.00	1.00
AG	171 (52.3%)	56 (43.6%)		1.00 (0.58-1.70)	0.72 (0.39-1.34)
AA	77 (23.5%)	41 (33.6%)		1.62 (0.90-2.90)	1.27 (0.65-2.48)
GG^d^	79 (24.2%)	26 (22.8%)	0.500	1.00	1.00
GA/AA	248 (75.8%)	97 (77.2%)		1.19 (0.72-1.96)	1.13 (0.63-2.00)
GG/GA^d^	250 (76.5%)	82 (66.7%)	0.035^a^	1.00	1.00
AA	77 (23.5%)	41 (33.3%)		1.62 (1.03-2.56)	1.59 (0.93-2.70)
**rs3829078**					
AA^d^	297 (90.8%)	114 (92.7%)	0.717	1.00	1.00
AG	29 (8.9%)	9 (7.3%)		0.81 (0.37-1.76)	0.84 (0.34-2.06)
GG	1 (0.3%)	0 (0%)		u.a.	u.a.
AA^d^	297 (90.8%)	114 (92.7%)	0.533	1.00	1.00
AG/GG	30 (9.2%)	9 (7.3%)		0.78 (0.36-1.70)	0.79 (0.32-1.93)
AA/AG^d^	326 (99.7%)	123 (100%)	0.539	1.00	1.00
GG	1 (0.3%)	0 (0%)		u.a.	u.a.
**rs1048638**					
CC^d^	292 (89.3%)	98 (79.7%)	0.011^a^	1.00	1.00
CA	33 (10.1%)	25 (20.3%)		2.26 (1.28-3.98)	1.98 (1.03-3.79)
AA	2 (0.6%)	0 (0%)		u.a.	u.a.
CC^d^	292 (89.3%)	98 (79.7%)	0.007^a^	1.00	1.00
CA/AA	35 (10.7%)	25 (20.3%)		2.13 (1.21-3.73)	1.92 (1.01-3.65)
CC/CA^d^	325 (99.4%)	123 (100%)	1.000	1.00	1.00
AA	2 (0.6%)	0 (0%)		u.a.	u.a.
**376deletion393**					
Ins/Ins^d^	249 (76.1%)	96 (78.0%)	0.651	1.00	1.00
Ins/del	76 (23.3%)	27 (22.0%)		0.92 (0.56-1.52)	0.86 (0.47-1.57)
Del/del	2 (0.6%)	0 (0%)		u.a.	u.a.
Ins/Ins^d^	249 (76.1%)	96 (78.0%)	0.671	1.00	1.00
Ins/del or del/del	78 (23.9%)	27 (22.0%)		0.90 (0.55-1.48)	0.84 (0.46-1.52)
Ins/Ins or ins/del^d^	325 (99.4%)	123 (100%)	1.000	1.00	1.00
Del/del	2 (0.6%)	0 (0%)		u.a.	u.a.

Statistical analysis: logistic regression model or chi-square or Fisher's exact tests, ^a^*p* < 0.05.

^b^Comparison between patients with uterine cervical cancer and control women.

^c^The adjusted OR with their 95% CI was estimated by logistic regression models after controlling for age between cancer patients and control women.

^d^Used as a reference for comparison to evaluate the odds ratio of other genotypes.

OR, odds ratio; 95% CI, 95% confidence interval. Del, deletion; ins, insertion; u.a., unavailable.

### Analysis of allele frequencies of CA9 polymorphisms in women with cervical cancer and controls

The minor allele frequencies of *CA9* SNPs analyzed in this study were 0.49 for rs2071676, 0.05 for rs3829078, and 0.06 for rs1048638, which are similar to those in HCB recorded in the National Center for Biotechnology Information SNP database. Only the mutant allele A in the *CA9* SNP rs1048638 tended to increase the risk of cervical cancer (*p* = 0.017; AOR: 1.75; Table 2). Other *CA9* SNPs and 376del393 did not carry this risk.

**Table 2:** Allele distribution of single nucleotide polymorphisms (SNPs) of carbonic anhydrase 9 in patients with uterine cervical cancer or normal women

Variables	Normal (n=327)	Cervical cancer(n=123)	*p* value	OR (95% CI)^b^	Adjusted OR(95% CI)^c^
**rs2071676**					
G^d^	329	108	0.087	1.00	1.00
A	325	138		1.29 (0.96-1.74)	1.16 (0.82-1.63)
**rs3829078**					
A^d^	623	237	0.483	1.00	1.00
G	31	9		0.76 (0.36-1.63)	0.75 (0.32-1.80)
**rs1048638**					
C^d^	617	221	0.017^a^	1.00	1.00
A	37	25		1.89 (1.11-3.21)	1.75 (0.95-3.22)
**376deletion393**					
Insertion^d^	574	219	0.604	1.00	1.00
Deletion	80	27		0.89 (0.56-1.41)	0.83 (0.47-1.44)

Statistical analysis: logistic regression model or chi-square test, ^a^*p* < 0.05.

^b^Comparison between patients with uterine cervical cancer and control women.

^c^The adjusted OR with their 95% CI was estimated by logistic regression models after controlling for age between cancer patients and control women.

^d^used as a reference to evaluate the odds ratio. OR, odds ratio; 95% CI, 95% confidence interval.

### Role of microRNA in the CA9 SNP rs1048638

The mutant allele A in the *CA9* SNP rs1048638 was a crucial factor for cervical cancer in the analysis of the genotype and allele distribution in Taiwanese women. We hypothesize that microRNAs (miRNAs) interact with the 3′-UTR of exon 11 of *CA9*, where the *CA9* SNP rs1048638 is located. We analyzed the *CA9* 3′-UTR in miRNA databases by using miRanda and TargetScan for targeting *CA9* miRNAs and found that rs1048638 was located in the target sequence of miR-34a. These results were further supported by the results of assessing the optimal minimal free energy duplexes of miR-34a and *CA9* 3′-UTRs. The A allele of *CA9* rs1048638 led to a substantially lower energy (−12.9 kcal/mol) than the C allele (−17.4 kcal/mol) with miR-34a hybridization, as previously described [23]. Collectively, these results suggest that miR-34a inhibits *CA9* expression by binding to the predicted binding sites on the 3′-UTR of exon 11 of *CA9* mRNA with the wild type C but not the mutant A in the *CA9* SNP rs1048638.

### Elevated CAIX expression associated with increased cell migration and invasiveness in cervical cancer cells

**Figure 1:**
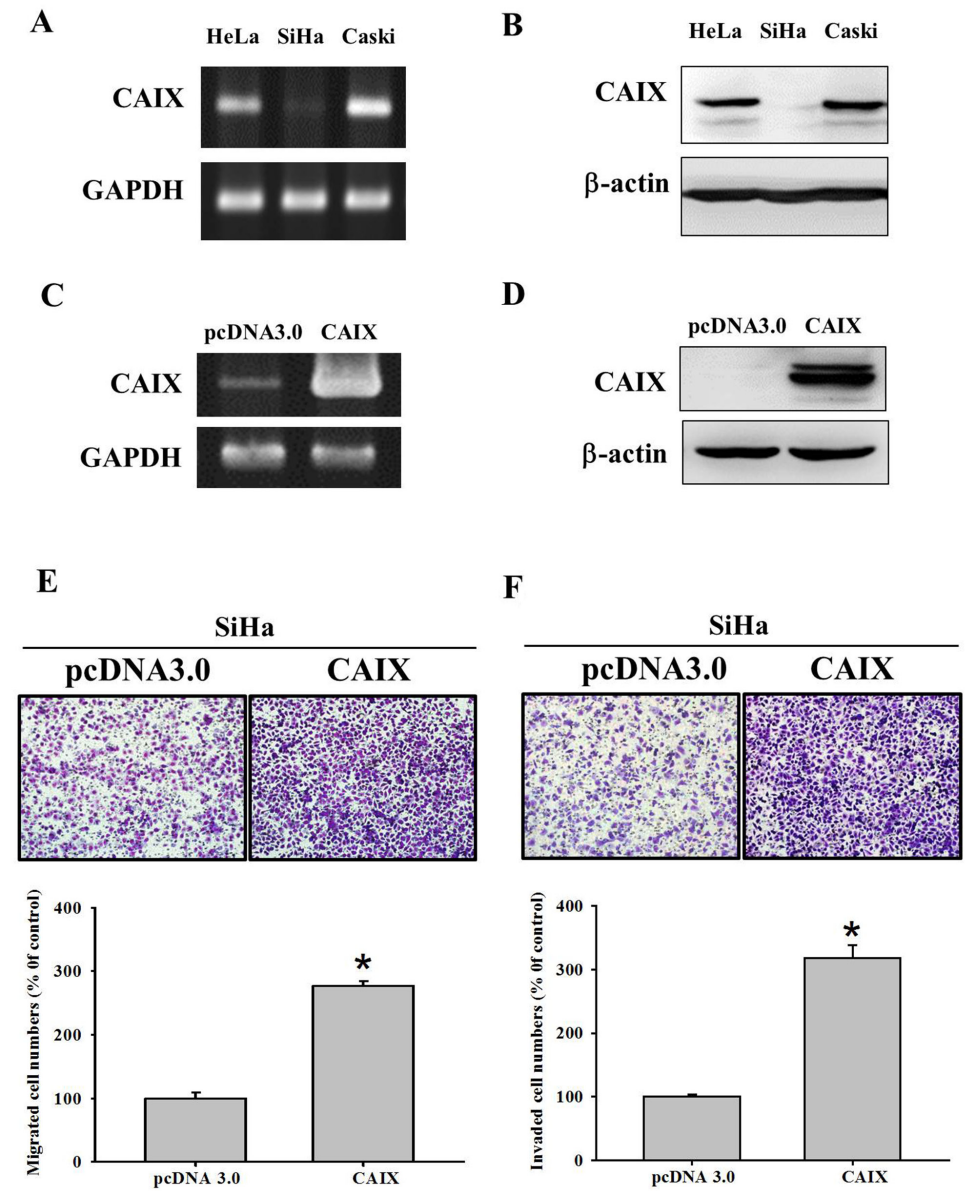
The mRNA and protein expressions of carbonic anhydrase IX (CAIX) in different cancer cell lines **(A)** The mRNA and **(B)** protein expressions of CAIX in SiHa cervical cell lines were lowest among SiHa, HeLa and Caski cancer cell lines of uterine cervix. The **(C)** mRNA and **(D)** protein expressions of CAIX were elevated in SiHa cell lines with CAIX gene transfection. The mRNA and protein expressions of CAIX were detected by RT-PCR and Western blot analysis. GAPDH and β-actin were separately used as internal controls. The **(E)** migratory and **(F)** invasive abilities of pcDNA3.0 and pcDNA3.0-CAIX SiHa cells were evaluated using Boyden chamber migration and Matrigel invasion assays. Differences are presented as the mean of triplicate experiments compared with control cells. *p < 0.05 compared with control cells.

To confirm the importance of the rs1048638 polymorphism in the regulation of *CA9* expression by miR-34a, we examined the rs1048638 genotypes of three cervical cancer cell lines (HeLa, SiHa, and Caski). The mRNA expression of *CA9* was the highest in Caski and the lowest in SiHa cervical cancer cells (Figure 1A). Similarly, the protein expression of CAIX was the highest in Caski and the lowest in SiHa cell lines (Figure 1B). Therefore, the overexpression of *CA9* in SiHa cervical cancer cells was analyzed. After *CA9* transfection, the mRNA and protein expression of CAIX was confirmed using reverse transcription polymerase chain reaction and a Western blot assay (Figure 1C and 1D). The overexpression of CAIX significantly increased the migratory and invasive ability of SiHa cell lines (Figure 1E and 1F). These results indicate a functional role of CAIX in promoting cell motility in cervical cancer.

### Influence of CAIX expression on cell migration and invasiveness through miR-34a in cervical cancer cells

**Figure 2:**
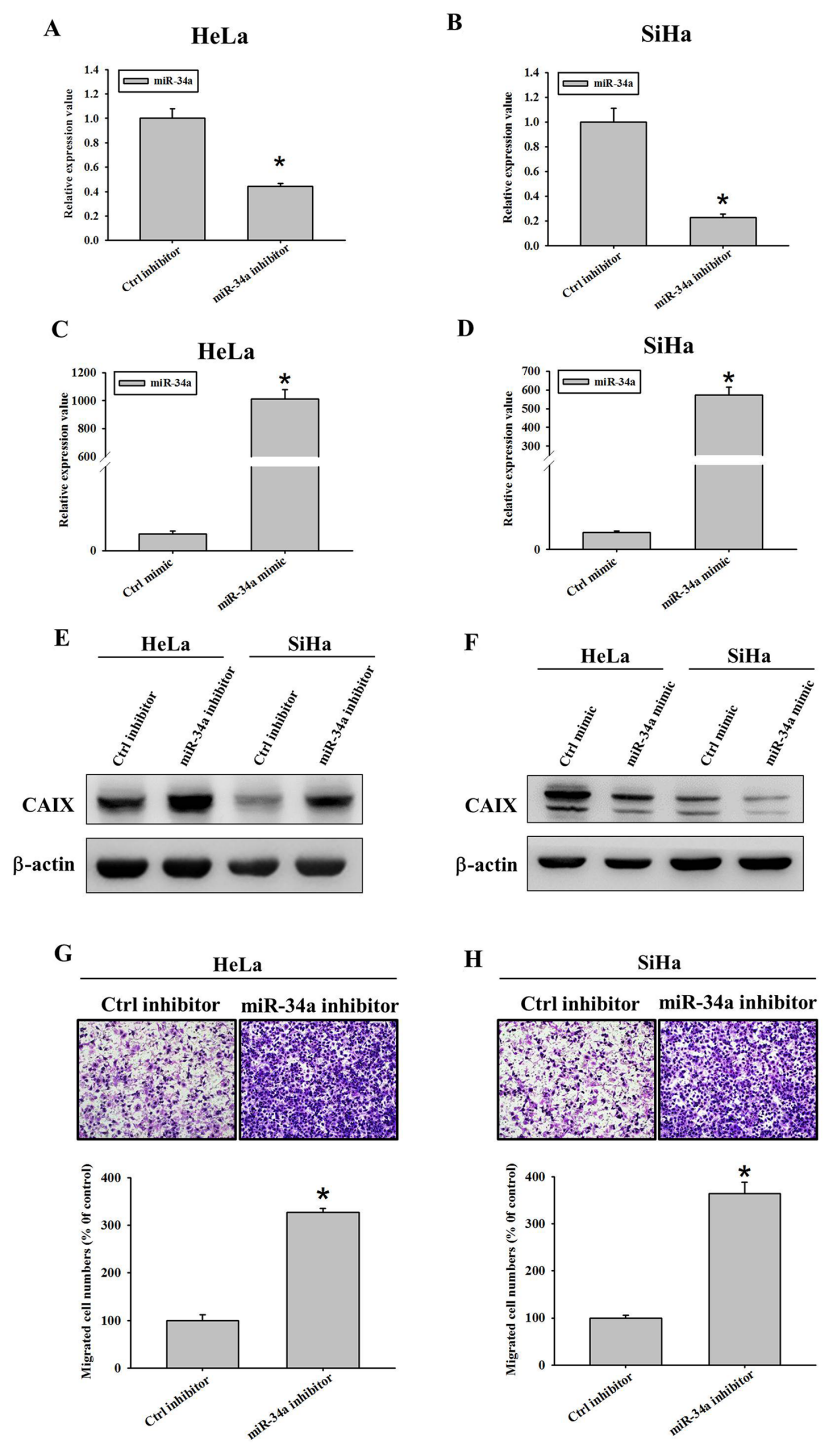
Effect of miR-34a inhibitor and mimic on the protein expressions of carbonic anhydrase IX (CAIX), migratory and invasive abilities in different cancer cell lines Reduced expressions of miR-34a after miR-34a inhibitor transfection into **(A)** HeLa and **(B)** SiHa cancer cell lines of uterine cervix. Elevated expressions of miR-34a after miR-34a mimic transfection into **(C)** HeLa and **(D)** SiHa cancer cell lines. The RNA levels were determined by Real time PCR 24 hours after miR-34a mimics and inhibitors transfection into these cancer cells. **(E)** Elevated expressions of CAIX protein after 200 nM miR-34a inhibitor transfection into HeLa and SiHa cancer cell lines of uterine cervix. **(F)** Reduced expressions of CAIX protein after 100 nM miR-34a mimic transfection into HeLa and SiHa cancer cell lines. The protein levels were determined by Western blot 24 hours after miR-34a inhibitors and mimics transfection into these cancer cells. **(G-H)** Increased migratory abilities in (G) HeLa and (H) SiHa cervical cancer cells with miR-34a inhibitor, as compared to their negative control cells without inhibitor by Boyden chamber assay.

After transfection of HeLa and SiHa cervical cancer cells with an miR-34a mimic and miR-34a inhibitor, the expression of miR-34a was determined to be increased and reduced, respectively, in these cancer cells by using real-time polymerase chain reaction (*p* < 0.001 and *p* < 0.001 for HeLa cells, Figure 2A and 2B; *p* < 0.001 and *p <* 0.001 for SiHa cells, Figure 2C and 2D). Moreover, after transfection of HeLa and SiHa cervical cancer cells with the miR-34a inhibitor, the expression of CAIX was elevated (Figure 2E). By contrast, after transfection with the miR-34a mimic, the expression of CAIX was reduced (Figure 2F). We further evaluated the influence of the miR-34a inhibitor on the migration and invasiveness of HeLa and SiHa cells and found them to be increased (Figure 2G and 2H). This result indicates that the miR-34a inhibitor may inhibit the suppression of CAIX expression by miR-34a and then increase the expression of CAIX. Consequently, the migration and invasiveness ability were elevated in both cancer cell lines.

### Association between CAIX expression and clinicopathological variables, cancer recurrence, and survival of patients with cervical cancer

The H score of the CAIX immunoreactivity of cancer tissues in 149 patients with cervical cancer was significantly higher than that of normal tissues in 29 normal controls (median: 0.5 vs. 0; *p* = 0.012). Patients whose tissues exhibited higher H scores of CAIX immunoreactivity had a more advanced stage (>stage I) compared with those with lower H scores (*p* = 0.014; Table 3). Nonsignificant associations between higher H scores of CAIX and higher recurrence probability and between higher H scores and shorter survival (*p* = 0.630 and *p* = 0.197; based on Kaplan–Meier curves) were observed.

**Table 3:** The association of carbonic anhydrase IX (CAIX) immunoreactivity in 149 cancer tissue microarrays with clinicopathological parameters of uterine cervical cancer

Clinicopathological variables^b^	CAIX^c^		*p* values	OR and 95% CI
	(+)	(-)		
Stage			0.014^a^	
I	8	78		1.00
Others (II + III + IV)	12	36		3.25 (1.10-9.94)
Pathologic type			1.000	
squamous cell carcinoma	22	99		1.00
adenocarcinoma	4	21		0.86 (0.19-2.92)
Depth of stromal invasion			0.913	
≤10 mm	7	46		1.00
>10 mm	10	62		1.06 (0.33-3.54)
Tumor grade			1.000	
well	3	17		1.00
moderate or poor	19	85		1.27 (0.32-7.40)
Parametrial invasion			0.576	
no invasion	19	93		1.00
invasion	7	26		1.32 (0.42-3.73)
Vaginal invasion			0.232	
no invasion	20	103		1.00
invasion	6	16		1.93 (0.55-6.01)
Pelvic lymph node metastasis			0.161	
negative	18	97		1.00
positive	8	22		1.96 (0.65-5.49)

Statistical analysis: chi-square or Fisher's exact test,

^a^*p* < 0.05.

^b^Some clinicopathological data could not be collected from the patients with cervical cancer due to incomplete records of medical charts.

^c^(+): positive immunoreactivity; (-): negative immunoreactivity. The median value of all H scores of CAIX in 149 cervical cancer cores was determined as the cutoff point to separate CAIX positive from CAIX negative tissue cores. Semiquantitative H score of CAIX immunoreactivity was calculated by multiplying the proportion score of stained cells by their immunoreactivity intensity.

OR: odds ratio; CI: confidence interval.

## DISCUSSION

In our study, we demonstrated that the genotype CA/AA increases the susceptibility of Taiwanese women to cervical cancer by using the wild homozygous genotype CC as a reference in the *CA9* SNP rs1048638. In addition, the mutant homozygous genotype AA was demonstrated to increase the susceptibility of Taiwanese women to invasive cervical cancer by using GG/GA as a reference in rs2071676. Furthermore, we observed that only one mutant allele A is sufficiently strong to increase the risk of cervical cancer in the *CA9* SNP rs1048638 based on the analysis of the allele distribution for *CA9* SNPs. However, two mutant alleles were necessary to increase the risk of cervical invasive cancer in the *CA9* SNP rs2071676. Paired allele mutation (homozygous mutant AA) was required for an increased risk of cervical cancer in comparison with GG/GA in the *CA9* SNP rs2071676.

Bioinformatics analysis was performed for the three SNPs, rs2071676, rs3829078, rs1048638, and the deletion mutation 376del393 in *CA9* in our study. The *CA9* SNP rs2071676 is located on exon 1 in chromosome 9p13.3, and a nucleotide change from G to the mutant A may lead to a nonsynonymous function (amino acid change from valine to methionine) [24]. This polymorphism is correlated with the signal peptide of CAIX and may influence its function [24]. The SNP rs3829078 is a known SNP of A1019G (Gln326Arg) located on exon 7 and also has a nonsynonymous function [24]. It affects the nearby substrate binding site of the CA active domain of CAIX [25, 26]. However, in our study, we did not identify a role of the SNP rs3829078 in the occurrence of cervical cancer. The SNP rs1048683 is located on the 3′-UTR region and near the *cis* elements of the human poly(A) site of chromosome 11, and an SNP in the 3′-UTR of a gene may influence biological processes [18, 24]. We found that the mutant allele A was involved in the development of cervical cancer. This SNP implication may result from miRNA–mRNA interaction. We found that two miRNAs, miR-34a and miR-449a, were involved in the rs1048638 site by using the TargetScanHuman prediction server. In the prediction of miRNA/target duplex interactions between miR-34a and *CA9* among different alleles, we found a 21.2% difference in minimum free energy between the C and A alleles by using the Bielefeld bioinformatics server [27, 28]. This explains the decreased binding of miR-34a with the 3′-UTR of exon 11 of *CA9*, where the *CA9* SNP rs1048638 is located, in women with cervical cancer with the mutant allele A in the *CA9* SNP rs1048638 and the elevated CAIX expression in Taiwanese women.

*CA9* 376del393 is a known deletion mutation of “DEEDLP” (“AspGluGluAspLeuPro”) in the proteoglycan domain and is located on chromosome 1. It may affect the proteoglycan domain of CAIX, which acts as an excellent catalyst for CO_2_ hydration at acidic pH values [29]. A distinctive feature of CAIX among all known CAs is the presence of a proteoglycan region of 58 amino acids, which is situated extracellularly at the amino-terminal region of the protein, in front of the CA catalytic domain [29]. This domain is rich in acidic amino acid residues with 8 aspartic acid and 18 glutamic acid residues constituting a total of 58 residues [30]. The presence of 26 COOH side chains from these acidic amino acid residues within the proteoglycan domain enables this domain to function in a buffer-like manner because the carboxylate/carboxylic acid conjugate bases are excellent biological buffers. The domain provides an intrinsic buffer promoting efficient CO_2_ hydration at acidic pH values, which is observed in hypoxic tumors. However, in our study, 376del393 did not exhibit a significant role in the susceptibility of Taiwanese women to cervical cancer. Our finding is in agreement with that of Chien et al. [20]. They reported that 376del393 is not associated with the susceptibility to oral cancer. Based on our findings that no patients with cervical cancer had 376del393 in both homologous chromosomes and those of de Martino et al. that the novel deletion 376del393 was present only in two patients with renal cell carcinoma [24], 376del393 appears to protect individuals from developing cancer.

In this study, cervical cancer tissues exhibited higher immunohistochemical expression of CAIX compared with normal tissues. Moreover, patients whose tissues exhibited higher H scores of CAIX immunoreactivity had more advanced stages (>stage I, beyond uterus) compared with those with lower H scores. Transfection with themiR-34a mimic led to changes in the miRNA–mRNA duplex interaction in the *CA9* SNP rs1048638 and reduced the CAIX expression, which reduced the migratory abilities of HeLa and SiHa cervical cancer cells. By contrast, the miR-34a inhibitor increased CAIX expression and therefore elevated its migration. Previous studies have demonstrated that miR-34a contributes to the development and progression of cervical cancer [31–33]. Moreover, Imani et al. demonstrated that miR-34a inhibits breast cancer cell migration by targeting epithelial-to-mesenchymal transition–inducing transcription factors [34]. In addition, the overexpression of CAIX in parental SiHa cells resulted in a significant elevation of the migratory and invasive abilities of SiHa cervical cancer cells. However, no patient with higher CAIX immunoreactivity exhibited deeper stromal invasion or more lymph node metastasis. Furthermore, a nonsignificant association was observed between a higher H score and shorter survival. Woelber et al. demonstrated that enhanced CAIX expression is an important feature of carcinogenesis in cervical cancer [35]. In their analysis, moderate or strong intratumoral CAIX expression was significantly associated with an advanced tumor stage, a higher depth of invasion, an undifferentiated tumor grade, and a higher preoperative serum squamous cell carcinoma level. Furthermore, the patients with cervical cancer with advanced stages (>stage I; CAIX expression: moderate or strong: 63 patients, none or weak: 22) exhibited stronger CAIX expression (*p* = 0.027; we calculated the *p* value based on their data) than those with stage I cervical cancer (moderate or strong: 74 patients, none or weak: 62). In agreement with our findings, Woelber et al. could not find a significant association between CAIX expression and patient survival. High CAIX expression has been reported to be more frequent in patients with cervical cancer with lymph node metastasis [36, 37]. However, Liao et al. demonstrated that immunohistochemical CAIX expression is an independent prognostic factor for both progression-free survival and overall survival in patients with high-risk, early-stage cervical cancer who received adjuvant pelvic radiotherapy with or without radiosensitizing chemotherapy [38]. Our study has some limitations. First, it lacked animal investigation, which could provide additional support to our findings. Furthermore, the molecular functional role of the binding between *CA9* 3′-UTR mRNA and miR-34a binding in cervical cancer requires further investigation.

In conclusion, our study reveals that the *CA9* SNP rs1048638 is the only significant polymorphism that increases the risk of cervical cancer among four *CA9* gene polymorphisms, rs2071676 (+201, G/A), rs3829078 (+1081, A/G), rs1048638 (+1584), and an 18-base pair deletion/insertion, in Taiwanese women. We discovered that the *CA9* SNP rs1048638 influences the expression of *CA9* through the interaction between the 3′-UTR of exon 11, where the SNP is located, and miR-34a. Elevated expression of CAIX was observed after transfection of HeLa and SiHA cervical cancer cells with the miR-34a inhibitor, which increased the migratory abilities of these cells through the inhibition of the microRNA–mRNA (miR-34a with 3′-UTR containing allele C of *CA9* SNP rs1048638) duplex interaction. We demonstrated that CAIX immunoreactivity is related to the occurrence of uterine cervical cancer, and elevated CAIX immunoreactivity is associated with a more advanced stage of cervical cancer. These findings may be applicable to translational medicine.

## MATERIALS AND METHODS

### Population

Four hundred fifty Taiwanese women, including 123 patients with uterine cervical cancer and 327 control Taiwanese women, were consecutively recruited into this study. The studied subjects all live in Mid-Taiwan where our hospital is located. Patients with cervical cancer were clinically staged I (n=79) or others (stage II, III and IV; n=44) based on the International Federation of Gynecology and Obstetrics Classification and received routine protocols at the Department of Obstetrics and Gynecology in Chung Shan Medical University Hospital, Taiwan, between January 1, 1999 and August 31, 2010. The histological types of the 123 cervical cancer tissues included 93 squamous cell carcinoma and 30 adenocarcinoma. Their grading were 31 grade 1, 73 grade 2 and 19 grade 3. Meanwhile, 327 control women without previous cancer history, who had normal Papanicolaou smear and were further verified using colposcopy in general examination at outpatient department in Chung Shan Medical University Hospital, were enrolled. The ages of women with cervical cancer and normal women were 53.9 ± 11.9, and 44.2 ± 10.2 (mean ± SD) years old, respectively. The study was approved by Institutional Review Board, Chung Shan Medical University Hospital (CSMUH No: CS11152). Each participant completed the informed consent.

### Selection of CA9 gene polymorphisms

Over 30 SNPs have been documented in the 11 exons region of the *CA9* gene in NCBI (National Center for Biotechnology Information), database SNP (dbSNP). We selected one SNP rs2071676 (+201, G/A) in exon 1, one SNP rs3829078 (+1081, A/G) in exon 7, one SNP rs1048638 (+1584, C/A) in the 3’-UTR of exon 11 as well as one polymorphism of an 18-base pair deletion/insertion (376deltion393 in exon 1) of *CA9* gene based on Chinese HapMap (Han Chinese in Beijing, China) data and the studies of Chien et al. [20]. The minor allele frequencies of these polymorphisms are ≥ 5%.

### Blood samples collection and genomic DNA extraction

One hundred and twenty-three blood specimens were collected from patients with cervical cancer. Overall, 327 blood specimens were collected from control women. Genomic DNA was extracted from EDTA anticoagulated venous blood using a QIAamp DNA blood mini kits (Qiagen, Valencia, Valencia, CA, USA) as described in detail previously [39, 40]. DNA was dissolved in Tris ethylene buffer (10 mmol/L Tris and 1 mmol/L EDTA; pH 7.8), and thereafter quantified by a measurement of OD260. Final preparation was stored at -20°C and applied as a template in polymerase chain reaction (PCR).

### Determination of single nucleotide polymorphisms of CA9 using real time polymerase chain reaction and genotyping

The allelic discrimination of the *CA9* SNPs rs2071676 (Assay ID: C_25472146_10), rs3829078 (Assay ID: C_27507259_10) and rs1048638 (Assay ID: C_1294917_10) was assessed with the ABI StepOne Real-Time PCR System (Applied Biosystems, Foster City, CA, USA), and analyzed by the TaqMan assay using SDS vers. 3.0 software as previously described [20]. The 376del393 SNP was detected with PCR. The PCR products were then electrophoresed through 3% agarose gels and stained with ethidium bromide. The primer sequences and probes for analysis of the *CA9* gene polymorphisms were described in our previous study [20].

### The influence of CA9 SNP rs1048638 on the immunohistochemical expression of CAIX protein and the clinical implication of CAIX protein in cervical cancer based on the tissue microarrays

In order to detect the influence of *CA9* SNP rs1048638 on the expression of CAIX protein, we simultaneously collected the tissue and blood samples to relate the allele distribution of *CA9* SNP (+1584, C/A) with the immunohistchemical expression of CAIX from 25 patients among 123 patients with cervical cancer, whom we recruited previously. Moreover, we established cervical tissue microarrays, consisting of normal and cancer tissues, to evaluate the clinical implication of CAIX in cervical cancer. Tissue microarray sections (MaxArray tissue cores, Zymed Laboratories Inc.) were incubated with anti-CAIX antibody (1:300 dilution; Novocastra^TM^ Liquid Mouse Monoclonal Antibody Carbonic Anhydrase IX, Product Code: NCL-L-CAIX; Newcastle Upon Tyne NE12 8EW United Kingdom). A semi-quantitative H score of CAIX immunoreactivity was determined by multiplying the proportional score of stained cells by their immunoreactivity [41–44].

### Isolation of RNA, reverse transcription-polymerase chain reaction (RT-PCR), quantitative real time PCR and western blotting

Total cellular RNA was extracted from SiHa, HeLa and Caski cervical cancer cells using RareRNA (Genepure Technology, Taiwan) according to the manufacturer's protocol. For PCR amplification, the primer sets were described in our previous study [14]. For western blotting, an equivalent amount of total protein was processed by 12% SDS-PAGE and electroblotted onto Hybond ECL PVDF membranes, and incubated with primary antibodies against human CAIX (1:1000 dilution; Novocastra^TM^ Liquid Mouse Monoclonal Antibody Carbonic Anhydrase IX) and β-actin (Sigma, St. Louis, MO, A5441).

### The influence of CAIX expression on the migration and invasiveness of cervical cancer cells

Cell migration and invasiveness assays were performed using modified Boyden chambers with 25 × 80 mm porous (12 μm size) polycarbonate membrane or 10× Matrigel precoated polycarbonate membrane (for cell invasiveness) using a 48 well chamber from Neuro Probe, Inc. The cells, which passed through the membrane, were stained with 20% Giemsa stain (Merck, Darmstadt, Germany) as previously described [14]. The cells on per well were in full photograph in microscopic fields at a magnification of ×40 and then were counted for the total migrated or invasive cells in triplicate.

### Statistical analysis

The independent Student's *t* test was used to analyze the age distribution of studied population, including patients with cervical cancer and control women. Chi-square or Fisher's exact tests were used to examine the relationships among frequencies of *CA9* gene SNPs or allele and incidence of cervical cancer between patients with cervical cancer and control women. Logistic regression model was used to analyze the comparisons of SNPs genotypes or alleles of *CA9* gene after controlling the age variable. The Mann-Whitney test was used to compare the CAIX expression between cervical cancer tissues and normal tissues as well as between cancer tissues of cervical cancer patients with wild homozygous *CA9* SNP rs1048638 (CC) and those with heterozygous SNP (CA). Chi-square or Fisher's exact tests were also used to associate the expression of CAIX protein (higher or lower H score) with various clinicopathological parameters, such as clinical staged I or others (stage II, III and IV), histopathologic types including squamous cell carcinoma (SCC) or adenocarcinoma, cell grading (well and moderate or poor differentiation), invasion depth of cervical stroma (≤ 10 mm or>10 mm stromal invasion depth), parametrium and vagina invasion and lymph node metastasis found during radical abdominal hysterectomy in cervical cancer patients. The median H score of CAIX immunoreactivity of cervical cancer tissues was selected as a cutoff level to separating the cancer tissues with higher expression (strong staining) from those with lower expression (weak staining). Kaplan-Meier curves were plotted for the cervical cancer patients based on the CAIX expression for the probability of recurrence or overall survival between primary surgery and death or recurrence or the end of the study (May 31, 2012). Odds ratio (OR) and adjusted odds ratio (AOR; controlling for age) and their 95% confidence interval (CI) were calculated by WinPepi software, version 10.0. or SPSS, version 12.0. A significant difference was defined by *p* < 0.05.

## Abbreviations

CAIX: Carbonic anhydrase IX
HSIL: High squamous intraepithelial lesions
LSIL: Low squamous intraepithelial lesions
SNP: Single nucleotide polymorphism
UTR: Untranslated region
